# Tailoring the Surface Morphology and the Crystallinity State of Cu- and Zn-Substituted Hydroxyapatites on Ti and Mg-Based Alloys

**DOI:** 10.3390/ma13194449

**Published:** 2020-10-07

**Authors:** Konstantin A. Prosolov, Vladimir V. Lastovka, Olga A. Belyavskaya, Dmitry V. Lychagin, Juergen Schmidt, Yurii P. Sharkeev

**Affiliations:** 1 Institute of Strength Physics and Materials Science of SB RAS, Academicheskii Prospect 2/4, 634055 Tomsk, Russia; vladimirlastovka1948@gmail.com (V.V.L.); obel@ispms.tsc.ru (O.A.B.); sharkeev@ispms.tsc.ru (Y.P.S.); 2 Department of Mineralogy and Geochemistry, National Research Tomsk State University, Lenin Avenue, 36, 634050 Tomsk, Russia; dvl-tomsk@mail.ru; 3 Department of Electrochemistry, Innovent Technology Development, Pruessingstrasse 27 B, D-07745 Jena, Germany; JS@innovent-jena.de; 4 Research School of High-Energy Physics, National Research Tomsk Polytechnic University, Lenin Avenue, 30, 634050 Tomsk, Russia

**Keywords:** RF-magnetron sputtering, calcium phosphate, TEM, annealing, biomaterials

## Abstract

Titanium-based alloys are known as a “gold standard” in the field of implantable devices. Mg-based alloys, in turn, are very promising biocompatible material for biodegradable, temporary implants. However, the clinical application of Mg-based alloys is currently limited due to the rapid resorption rate in the human body. The deposition of a barrier layer in the form of bioactive calcium phosphate coating is proposed to decelerate Mg-based alloys resorption. The dissolution rate of calcium phosphates is strongly affected by their crystallinity and structure. The structure of antibacterial Cu- and Zn-substituted hydroxyapatite deposited by an radiofrequency (RF) magnetron sputtering on Ti and Mg–Ca substrates is tailored by post-deposition heat treatment and deposition at increased substrate temperatures. It is established that upon an increase in heat treatment temperature mean crystallite size decreases from 47 ± 17 to 13 ± 9 nm. The character of the crystalline structure is not only governed by the temperature itself but relies on the condition such as either post-deposition treatment, where an amorphous calcium phosphate undergoes crystallization or instantaneous crystalline coating growth during deposition on the hot substrate. A higher treatment temperature at 700 °C results in local coating micro-cracking and induced defects, while the temperature of 400–450 °C resulted in the formation of dense, void-free structure.

## 1. Introduction

Recently, medical devices are an inherent part of modern medicine. The constant demand for new, prospective implants is caused by global population aging [1]. The total number of patients worldwide subjected to the risk of bone fracture has been projected to double in 2040 [2]. Up until now, titanium (Ti) based alloys remain the most widely used materials for permanent implants when treating bone. Ti-based alloys are well studied and frequently being classified as the “gold standard” in metallic implants in both orthopaedic and dental areas. However, it is also documented that under the influence of mechanical stress-protective TiO_2_ layer could be disturbed and metallic particle leakage could be the reason for various adverse reactions in host tissue [3]. Magnesium (Mg) based alloys, however, are very promising biocompatible materials for biodegradable, temporary implants [4]. This material is readily bioresorbable in the body, releasing the Mg ions, which has a functional role in many physiological processes [5]. Most importantly, implants made of Mg do not require additional surgical intervention as they would be completely dissolved upon bone healing or at least intended to do so. Unfortunately, the main limiting factor for the widespread use of Mg-based implants is their fast degradation in the human body, when compared to bone tissue growth [6]. Besides, excessive production of hydrogen around the Mg-based implant due to its decomposition hinders successful bone healing. Many scientific groups are concerned about doping Mg with various elements to decelerate degradation rate or improve materials service characteristics [7,8]. One of the approaches is calcium alloying that helps to adjust the degradation rate, biocompatibility, and microstructure [8,9,10].

It should be noted that the market for antibacterial surfaces experienced steady growth over the recent years. World Health Organization reported that 60% of the patients suffered from nosocomial infections in the post-implantation period. One of the strategies to diminish the risk of peri-implantitis caused by unwanted bacteria in the implantation site is antibacterial coating deposition. Ideally, the deposited coating not only should possess an antibacterial effect, but also increase bone healing and improve osseointegration. From that perspective, substituted hydroxyapatites (HA) are one of the most promising materials for biomedical coatings [11,12,13]. It has been shown that inorganic antibacterial metallic ions (e.g., Cu, Zn, and Ag) can be introduced into the HA lattice and improve its antibacterial properties [14,15,16,17,18,19,20]. For instance, the effect of HA coatings containing 10% Zn and Mg on titanium implants were analyzed, and all of the coatings showed noticeable improvements in bone formation after 12 weeks of exposure in female rats. They also improved mechanical strength and osseointegration [21]. In our previous report, the antibacterial properties for a Cu and Zn containing HA coating on Ti were confirmed against the pathogenic strain 209P of Staphylococcus aureus [22].

Moreover, such a coating could be used as a protective substrate layer, limiting the degradation rate of Mg-based alloys and improving biological response to Ti-based materials [23]. For example, in the work by M. Sedelnikova et al. it was found that the Sr-substituted HA and Sr-substituted tricalcium phosphate coatings performed a protective function and reduced the corrosion rate of the Mg–0.8 wt % Ca alloy [24].

There are various methods to deposit calcium phosphates (CaP) such as thermal spraying techniques [25,26], micro-arc oxidation [27], various physical vapor deposition (PVD) techniques [28,29,30], wet chemical deposition [31], etc. Each of the mentioned techniques has its advantages and drawbacks, however, radiofrequency (RF) magnetron sputtering of CaP, a part of the PVD technique, remain attractive for the possibility to manipulate coatings parameters such as fine-tuning of surface morphology, crystallinity, elemental composition, and thickness in accordance to the desired values [32]. Moreover, successful examples of CaP coating deposition by an RF magnetron sputtering to slow down the Mg-based substrate corrosion rate are already known. For example, in the recent study by M.A. Surmeneva et al. [33] the ultrathin as-deposited CaP coating significantly enhanced the degradation resistance of Mg–1 wt.% Ca alloy. It is well-known that the degradation rate of CaP not only dependent on Ca/P ratio but on a material crystallinity state. It is well documented that amorphous CaP (ACP) coatings are more resorbable than crystalline. Hence, by deposition of CaP coatings with various crystallinity levels on the Mg-based alloys, it is possible to tailor the resulting implant corrosion rate, which might be useful in various clinical applications. In the following report [34] by M.A. Surmeneva et al. corrosion resistance of as-deposited and heat-treated (HT) at 400 °C CaP coatings Mg-based alloy (AZ91) has been investigated. Even though improved crystallinity of CaP coating should have increased the corrosion resistance, the results showed that HT did not significantly change the stability of a composite due to the treatment-induced microcracking. Therefore, it is essential to perform an HT in a way that the coating integrity remains.

Besides the CaP degradation rate, it is not yet clear what level of crystallinity is beneficial for the desired cell response and finally to osseointegration. ACP coatings application in treatment remains somewhat a controversial topic as reports supporting the beneficial effect of ACP experiments in vivo [35,36] contradicts published results concerning the negative impact of ACP on cells during studies in vitro [37]. Lastly, we should mention that it is also essential to control coatings topology and micro/nano- roughness as it is a major signalling system for cells. Various scientific groups recently have become concerned in developing structured patterned surfaces for biomedical applications [38,39,40].

The purpose of our study was to reveal the difference in crystallization, structure, and surface morphology of X-ray amorphous Cu and Zn containing calcium phosphates deposited by an RF magnetron sputtering on Mg–Ca and Ti alloys during post-deposition heat-treatment vs. deposition at increased substrate temperatures.

## 2. Materials and Methods

Commercially pure titanium samples (99.58 Ti, 0.12 O, 0.18 Fe, 0.07 C, 0.04 N, 0.01 H wt.%) of 10 × 10 × 1 mm^3^ size provided by “VSPO-AVISMA” (Perm, Russia) were used as substrates. Magnesium alloy Mg–0.8 wt.% Ca (Mg0.8Ca) that was also used as a substrate material in this study was developed at Helmholtz Zentrum (Geesthacht, Germany). The alloy was produced following the technology reported elsewhere [27]. In brief, the pure magnesium was molten in a mild steel crucible in the atmosphere of a gas mixture of argon and sulfur hexafluoride. Pure calcium was added, and the melt was stirred. After that, the melt was placed into a holding furnace and was kept at 720 °C for 1 h in the protective gas atmosphere of argon. The ingots were machined and cut for further use in the study. The surface of 10 × 10 × 1 mm^3^ substrates from Mg0.8Ca and Ti was ground with progressively finer grinding paper up to 2000 grit. After that, they were polished with a 15 µm diamond paste. Later, the samples were cleaned ultrasonically in distilled water within 10 min.

The sputtering targets were Cu- and Zn-substituted hydroxyapatite (Cu-HA and Zn-HA). A powder material was prepared by mechanochemical synthesis at the Institute of Chemistry and Mechanochemistry, Russian Academy of Sciences, Novosibirsk, Russia. The mechanochemical synthesis was carried out according to the reactions:

(1) $$6{\mathrm{CaHPO}}_{4}+\left(4-x\right)\mathrm{CaO}+\mathrm{xCuO}={\mathrm{Ca}}_{\left(10-x\right)}{\mathrm{Cu}}_{x}{\left({\mathrm{PO}}_{4}\right)}_{6}{\left(\mathrm{OH}\right)}_{2}+2H_{2}O,\;\mathrm{where}\;x\;=\;0.2$$

(2) $$6{\mathrm{CaHPO}}_{4}+\left(4-x\right)\mathrm{CaO}+\mathrm{xZnO}={\mathrm{Ca}}_{\left(10-x\right)}{\mathrm{Zn}}_{x}{\left({\mathrm{PO}}_{4}\right)}_{6}{\left(\mathrm{OH}\right)}_{2}+2H_{2}O,\;\mathrm{where}\;x\;=\;0.4$$

The prepared powder was used as a precursor for the preparation of a target for sputtering. The detailed description of production technology and experimental studies regarding the phase composition and structure are reported in our previous work [41]. Stainless steel press molds and the MIS-6000.4K hydraulic press (Armavir, Russia) were used for uniaxial pressing of obtained Cu-HA and Zn-HA powders. Targets of 110 mm in diameter were sintered in air using the ITM 12.1200 electric furnace (OOO “ITM”, Tomsk, Russia).

A vacuum installation, with a planar magnetron with a diameter of 110 mm, was operated at 13.56 MHz, in order to deposit the CaP coatings on metallic substrates. Only one type of samples made of either Ti or Mg0.8Ca alloy was used per deposition run. The metallic samples were mounted on the substrate holder with a built-in heater that was designed to reach a temperature of ~500 °C. An RF-power was ramped up to the working level values of 250 W in a step-wise manner (50 W per 15 min).

For the deposition of Cu-containing CaP coatings on a cold substrate, a Cu-HA target and substrates made of Ti and Mg0.8Ca alloy were used. The deposition time was 2 h, and the target-to-substrate distance was 70 mm. During the coating deposition, the working pressure in the vacuum chamber was controlled and was set to 0.1 Pa. The chamber base pressure before the deposition was 0.6 mPa.

For the deposition of Zn-containing CaP coatings on a hot substrate, a Zn-HA target and a substrate made of Ti were used. Four regimes of in-situ substrate heating were applied, being 100, 200, 300, and 400 °C. The substrate holder was heated in a step-wise manner with a heating rate of 50 min^−1^. The temperature of the samples was controlled by a chromel–alumel thermocouple. Pre-heated samples were transferred in a position under the target erosion. The argon gas pressure (base pressure 10^−5^ Pa, working pressure 0.7 Pa) and the distance between substrate and target (60 mm) were kept constant. The deposition time in the case of hot substrates and Zn-HA target was increased up to 3 h.

The surface morphology and composition of the coatings were analyzed using a scanning electron microscope (SEM) Tescan Mira 3 LMU (Brno, Czech Republic). Before SEM observation a conductive carbon layer of 15 nm thickness was deposited on the samples. The surface morphology of the deposited coatings was examined at a magnification of 50,000× and 100,000×. The as-deposited Cu-HA films in the case of cold substrates regime were found to be X-ray amorphous. We performed post-deposition heat treatment (HT) in order to obtain crystalline coatings. The heat treatment was performed in a quartz tube pumped to 1.3 Pa and filled with Ar gas up to the pressure of 60 kPa. The quartz tube was placed in a furnace, and the temperature gradient increase was programmed in a stepwise manner at 20 °C/min. Two separate set of experiments with heat treatment of 450 and 700 °C for a constant period of time set to three hours were performed.

The X-ray diffraction analysis for Cu-HA coatings on Ti substrates after HT was carried out with a Shimadzu XRD-6000 in CuKα-radiation (*λ* = 1.54 Å, 40 kV, 30 mA) in 2θ range of 25°–45° and the scanning step was 0.02°, preset time—1 s while measurements for Zn-HA coatings on Ti and Cu-HA coatings after HT on Mg0.8Ca were performed using DRON-7 (Burevestnik, Russia) in 2θ range from 15°–60°, radiation source being CoKα (*λ* = 1.7890 Å) and a scanning step of 0.01°. The measurements were carried out at constant rotation of substrates and using Bragg–Brentano geometry in all cases. The X-ray diffraction peaks of hydroxyapatite (#04-014-6258 and #09-432) and titanium (#00-044-1294) from ICDD PDF4+ database were used as references. An instrumental broadening was calculated from a standard Si powder. The method of full-profile analysis (Rietveld method) was employed for calculation of the lattice parameters, sizes of coherent scattering region, and internal stresses. Sizes of coherent scattering region (CSR) and internal stresses were estimated by the Scherrer equation:

*Bcos θ = 2Δd/dsinθ + 0.9λ/β* (3)

where *B* is full width on half maximum, *θ*—angle, Δ*d/d*—internal stresses, *λ*—wavelength (Cu) and *β*—the size of coherent scattering region [42].

In order to analyze the microstructure of the deposited Cu- and Zn-HA films, transmission electron microscope (TEM) JEM 2100 (JEOL, Tokyo, Japan) with in-column energy dispersive X-ray (EDX) INCA-Energy microanalyzer (Oxford Instruments, UK) was used. The distribution of Ca, P, O, Ti, Cu, and Zn in the coatings was determined by EDX in different regions across the lamella (results are reported in at.%). For the cross-section sample preparation an ion thinning equipment EM 09100IS ion slicer (JEOL, Japan) was used. To minimize the structural changes associated to sample preparation the final thinning was performed in low-energy and low-angle Ar-ion beam mode. TEM sample preparation and study were performed at the Center for Collective Use of Scientific Equipment “Nanotekh” of ISPMS SB RAS (Tomsk, Russia).

## 3. Results and Discussion

### 3.1. The Influence of P-Deposition Heat Treatment on the Structure of Cu-HA Deposited on Mg–Ca and Ti Substrates

**Figure 1:**
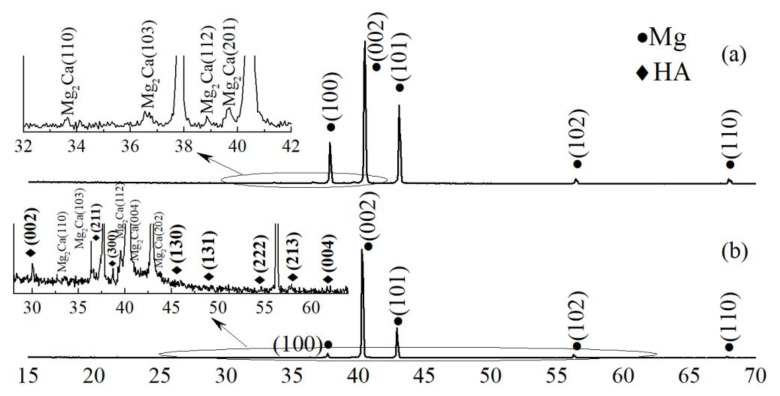
X-ray profiles of as-deposited Cu-hydroxyapatites (HA) coating on Mg0.8Ca (**a**) and Cu-HA coating on Mg0.8Ca after heat treatment (HT) at 450 °C for 3 h (**b**).

In our previous report [41], we analyzed an XRD data of a sintered Cu-HA target that was also used in the present study. The phase composition of a Cu-HA target was represented by a single phase of HA, with lattice parameters being: *a* = 0.9386 ± 0.0004 nm, *c* = 0.6895 ± 0.0005 nm, *c/a* = 0.7346 and *V* = 0.5261 ± 0.0003 nm^3^. As deposited coatings on the Mg-based substrate, however, did not reveal any peaks that could be attributed to CaP according to an XRD data (Figure 1a). In the range of 2θ = 15°–30° the region of diffuse scattering could be observed. The presence of such a region is suggesting the presence of an X-ray amorphous phase that could be attributed to CaP coating. The volume fraction of this region was estimated to be 2%. The well-defined peaks belong to the Mg phase while the peaks of small intensity are corresponding to Mg_2_Ca intermetallic phase. Both Mg and Mg_2_Ca phases belonged to the substrate material. The volume fraction of an intermetallic phase was 3% when the major fraction of 95 vol.% corresponds to an Mg phase.

The detailed analysis of the crystalline structure of HA coatings after annealing can be found in our previous report. An XRD profile of a post-HT sample with CaP coating is represented in Figure 1b. It was revealed that previously detected X-ray amorphous halo that was attributed to the CaP coating became fully crystalline after 450 °C HT with well-defined peaks. After heat-treatment, the coating exhibit a pure HA phase. The calculated lattice parameters of the coating material were *a* = 0.94443 ± 0.01218 nm and *c* = 0.69477 ± 0.001 nm. The CSR for the Cu-HA coating was 37 ± 15 nm. Also, annealing led to an increase of *a* and decrease of *c* lattice parameters for both phases corresponding to the substrate. As it is known from the structure zone theory models, a required (but not sufficient) condition for the appearance of amorphous phases is that the deposition temperature must be below 30% of the melting temperature of the material being sputtered. For higher values of temperature, the surface diffusion of deposited atomic species would allow for the formation of crystallites with long-range atomic order. The melting point of HA is 1650 °C. According to K. Tõnsuaadu et al. [43], ion substitution in HA lattice could cause a significant decrease in the melting point, and hence the temperature of recrystallization. In our case, the HT temperature of 450 °C allowed us to reach the coating crystallization, probably due to the Cu substitution in HA lattice. A significant increase in coating crystallinity was also reported in the work by Yang et al. [44] as the heating temperature was increased up to 450–500 °C.

**Figure 2:**
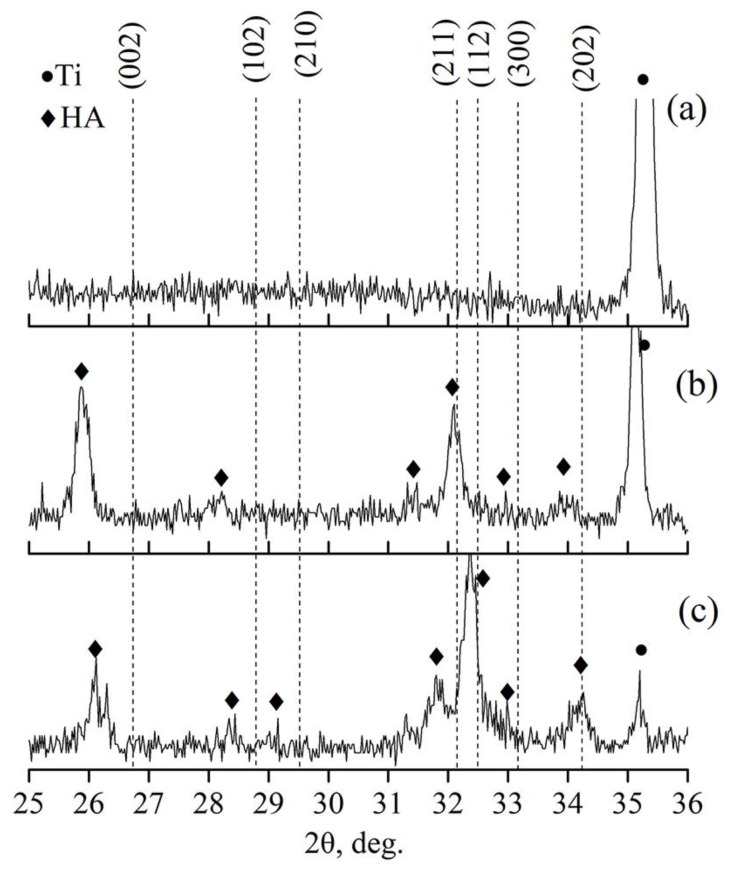
X-ray profiles of Cu-HA coating on Ti as-deposited (**a**), Cu-HA coating on Ti after annealing at 450 °C for 3 h (**b**) and Cu-HA coating on Ti after annealing at 700 °C for 3 h (**c**).

Cu-HA coating was also deposited on Ti substrates. As-deposited coatings were found to be an X-ray amorphous according to results depicted in Figure 2a. As the Ti substrate could tolerate higher treatment temperatures Cu containing ACP on Ti substrates has been HT at 450 and 700 °C for 3 h in the Ar atmosphere. According to X-ray profiles HT induced coating crystallization in both cases (Figure 2b,c). Orientation planes are plotted according to the HA standard values (ICDD PDF card. 04-014-6258) represented by dashed lines.

Upon an increase in HT temperature, preferential orientation was changed from (002) plane to (112) plane at 700 °C HT. Post-deposition HT at 700 °C resulted in an HA structure, although the XRD peaks were shifted towards higher 2θ angles, which is especially prominent in the 32°–33° region. This shift is due to the lattice parameters decrease upon the increase in HT temperature from 450 to 700 °C as it is seen from Table 1. Similar results regarding the crystal lattice decrease at increased HT temperatures have been reported by K. van Dijk [45]. The presence of TiO_2_ was not confirmed. It is probably due to the fact that the CaP protected the substrate from severe oxidation. Lattice parameters for Cu-HA coatings on both Ti and Mg0.8Ca substrates are represented in Table 1.

It is well documented that the lattice parameters are changed upon foreign ions substitution in HA lattice [43]. Therefore, we compare calculated lattice parameters of coatings with both standard values and those obtained experimentally from the Cu-HA target. Thus, the lattice parameter of Cu-HA coatings on Ti tends to decrease upon crystallization. When compared to both standard values and values for Cu-HA coating, lattice parameter c is lower than expected which is indicating the shrinkage in the *c*-axis for the unit cell. Cu-HA on Mg0.8Ca, however, shows an inverse relation. The Cu-HA coating on Mg0.8Ca after 450 °С HT has *c* = 0.6947 nm. This is probably induced by the difference in thermal expansion coefficient. For commercially pure Ti, a thermal expansion coefficient range from 7.6 × 10^−6^ to 9.8 × 10^−6^ /K [46], for HA this number is higher and equals to 15 × 10^−6^ /K [47], and the highest value is found for Mg0.8Ca alloy which equals to 25 × 10^−6^ /K [48]. Moreover, as average crystallite size is relatively close to one another for Cu-HA coatings on both Ti and Mg0.8Ca substrates at 450 °С HT, increased temperature on Ti facilitated the growth of finer crystallites due to rapid, competitive grain growth.

**Figure 3:**
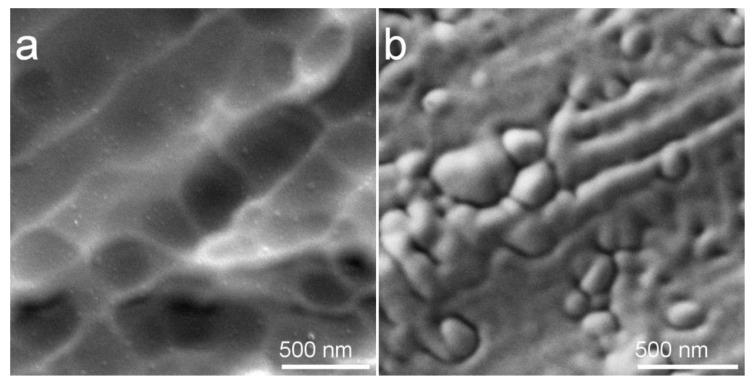
As-deposited Cu-HA coating on Mg0.8Ca (**a**) and on Ti substrate (**b**).

In Figure 3 SEM images of as-deposited Cu-HA coating on Mg0.8Ca obtained at an accelerating voltage of 10 kV and on Ti obtained at 3 kV is represented. In both cases, the coatings’ surface is dense and void-free which is essential for a barrier function. The surface is represented by a globular or dome-like surface features (Figure 3b). At higher accelerating voltage (Figure 3a), nanosized surface particulates are visible. It is known that the topographical irregularities and small foreign particles remaining on the surface of the substrate after cleaning or generated during the ion bombardment, cause the formation of growth defects in the coating due to the shadowing effect [49]. Moreover, various nodule types of defects could be present in thin films, however, their sizes are usually larger than observed 10–60 nm surface features in Figure 3a.

It could be so, that upon the sputtering process multiple bond destruction in HA crystals occur. Clusters of atoms, radicals, ions, and various Ca and P–O containing neutrals are traveling to the substrate after being sputtered from the surface of a target [50]. At low substrate temperature and relatively low energies, ACP is formed on the surface of the substrate. One can speculate that one of those clusters could be represented by a Ca_9_(PO_4_)_6_ (Posner cluster). Posner clusters are structural units of ACP, a prenucleation cluster of HA which has been proposed as a mineralization precursor with the size in the range of 1 nm having a globular-like form [51]. The abundant amount of globular-like surface features that do not appear to be nodular type defects of a thin film are visible in Figure 3 and could potentially be considered as agglomerates of ACP clusters.

**Figure 4:**
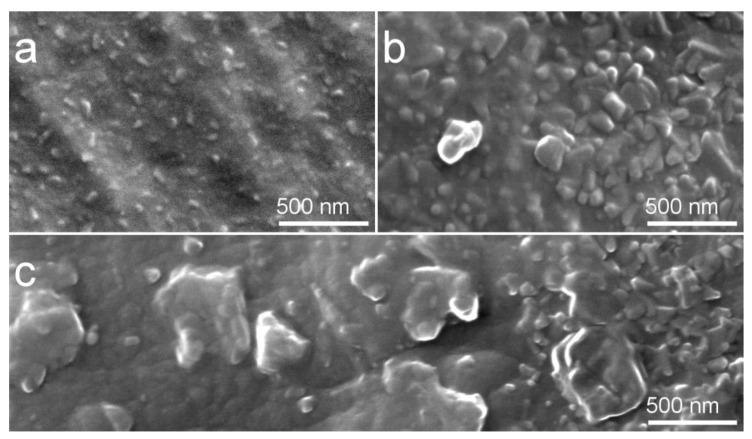
Scanning electron microscope (SEM) image of the Cu-HA coating on Mg0.8Ca alloy after HT at 450 °C showing fine crystallites (**a**) Cu-HA coating on Ti after HT at 700 °C showing the same type of crystallites of increased size(**b**) and Cu-HA coating on Ti after HT at 700 °C showing agglomerates of crystallites (**c**).

At the macroscale level, HT at 450 °C did not result in any evident cracking or heat-induced defects. Only at the magnification of 50,000×, the difference of as-deposited Cu-HA coating vs Cu-HA coating after 450 °C HT becomes evident (Figure 4). Post-deposition HT, in case of a lower temperature, did not result in coating delamination or cracking as evidenced by SEM. However, the Ca/P ratio was changed from 1.77 for as-deposited to 1.85 for the HT samples according to EDX measurements. An increase in Ca/P ratio due to heat-treatment is well documented [52]. Moreover, previously observed 10–60 nm-sized particulates are no longer seen, instead, we observed fine crystallites that are distributed across the whole sample. Moreover, at the increased HT temperature, on the surface of Ti, the same type of crystallites of increased size are seen in Figure 4b,c. In some region of the sample, crystallites are becoming agglomerates as it is seen in Figure 4c. Hence, we conclude that previously seen globular agglomerates of ACP clusters on as-deposited Cu-HA coatings acted like crystallization seeds that facilitated the crystallite growth. EDX measurements of Cu-HA on Ti substrates revealed that as-deposited coating had Ca/P ratio of 1.77 which was increased after 450 °C HT to 1.85 and further to 2.00 at 700 °C HT. Cu-HA coating HT at 700 °C resulted in cracking and coating bubbling when 450 °C HT did not result in any visible macroscopic change. Cu content varied from 0.1–0.6 at.% across the samples irrespective of HT temperature or substrate material.

**Figure 5:**
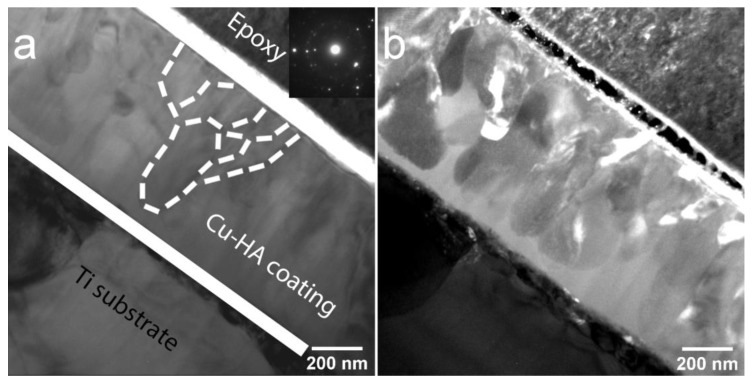
Transmission electron microscope (TEM) image of the cross-section of Cu-HA coating on Ti after HT at 450 °C bright field (BF), the inset shows the corresponding electron diffraction pattern (**a**) and dark field (DF) (**b**).

TEM analysis of bright field (BF) and dark field (DF) images accompanied by selected area electron diffraction (SAED) have been used to understand the local structure of the cross-section of the coating after 450 and 750 °C HT. In Figure 5, a TEM image a cross-sectional view of the Cu-HA coating on the Ti substrate after HT at 450 °C is represented. The structure of the Cu-HA coating is dense, no cavities or holes were observed. The mean coating thickness is 680 ± 8 nm. The contrast in the bright-field TEM image from the coating region is low, indicating that no precipitates occur and grains are weakly disorientated, grain boundaries are not pronounced. Here, and further on the dotted lines were hand-drawn for clarity of grains shape representation. SAED obtained from the coating region indicates the polycrystalline nature of the coating with a visible fraction of the amorphous phase which is confirmed by a weak diffusive halo, corresponding to the small fraction of the ACP phase. Overall, the sample appears to have a homogeneous fine structure. The columnar type of growth is evident from the TEM image with mostly elliptical shape of grains. The mean lateral grain size varies from 45 to 100 nm. This is well-correlated to the crystallite size obtained by XRD previously.

**Figure 6:**
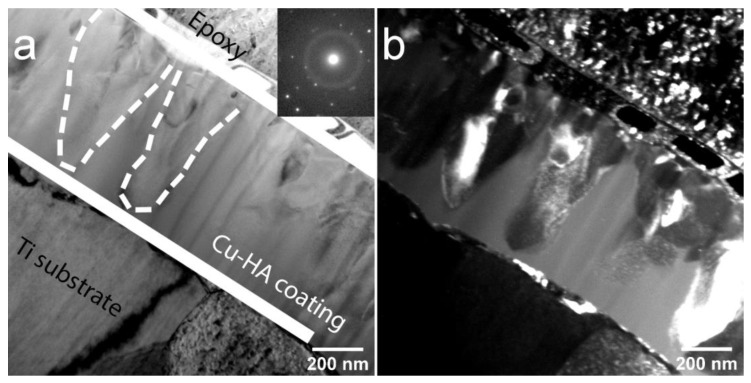
TEM image of the cross-section of Cu-HA coating on Ti after HT at 450 °C BF, the inset shows the corresponding electron diffraction pattern (**a**) and DF (**b**).

The crystallization did not occur homogeneously over the whole coating volume. In Figure 6a, a SAED shows a typical halo of an amorphous phase. In the DF image, distinct V-shaped grains that are consist of sub-grains finer in size are submerged in an amorphous matrix. The ACP is confirmed almost in all visible areas across lamella and situated near the interface between the crystalline part of a Cu-HA and the Ti substrate. From the obtained TEM images it could be concluded that the grain nucleation starts from the top side of the coating and propagates towards the Ti substrate is governed by the temperature diffusion gradient during HT. To understand the local composition, the EDX of elements was obtained from the cross-section of the coating. The sample is homogeneous and does not differ remarkably in compositions from one area to another, indicating the formation of homogenous CaP coating with a Ca/P ratio of 1.85. The precise Cu content in the EDX spectra is hindered due to the Cu sample holder used for the TEM analysis.

**Figure 7:**
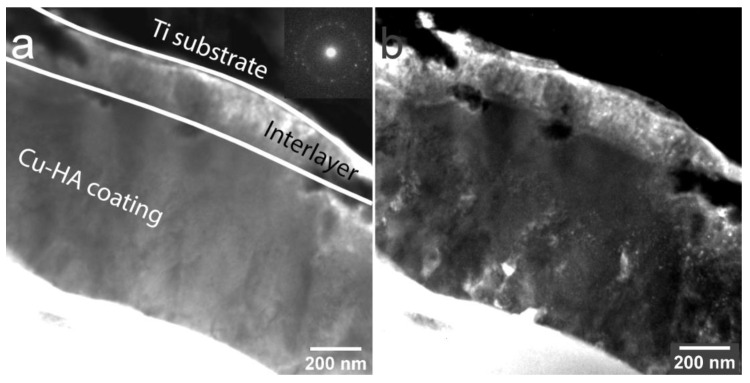
TEM image of the cross-section of Cu-HA coating on Ti after HT at 700 °C BF, the inset shows the corresponding electron diffraction pattern (**a**) and DF (**b**).

At increased HT temperature the microstructure of the coating is significantly changed (Figure 7). A distinct interlayer that consists of equiaxed grains is evident. The SAED reveals the polycrystalline structure of Cu-HA coating. No amorphous phase between the coating and the substrate was observed which is also supported by SAED.

**Figure 8:**
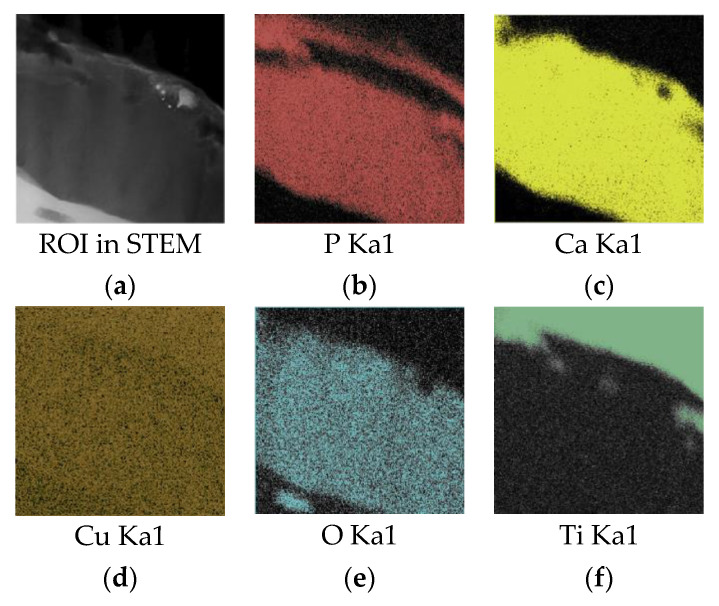
An energy dispersive X-ray (EDX) element mapping of the Cu-HA and Ti substrate in TEM lamella. (**a**) ROI in STEM; (**b**) P Ka1; (**c**) Ca Ka1; (**d**) Cu Ka1; (**e**) O Ka1; (**f**) Ti Ka1.

In column EDX mapping, from the region of interest (ROI) (Figure 8) it is revealed that after crystallization, phosphate groups (P) appear to be diffused in substrates surface sub-layer and in a columnar structured layer, leaving the equiaxed zone P-poor (Figure 8b). In the crystalline columnar region, a Ca/P ratio varied in the range 1.75–1.83, while in the equiaxed region only a trace amount of P could be found leaving the Ca/P ratio at around 11. The mean Ca/P ratio obtained during the mapping of the elements from the coating cross-section equals to 1.92. The concentration of elements in the Cu-HA coating according to in-column EDX is represented in Table 2.

According to the elemental composition, the formation of the CaO phase in the interlayer region is suggested. Similar results were obtained in the work of Kobayashi et al. [53] where the CaO phase was formed in the vicinity of the Ti substrate after as-sputtered amorphous coating was annealed at 600 °C. The formation of the interlayer was connected to the lack of P due to evaporation. However, in our case, diffusion of the P in the Ti sublayer region could not be neglected. A strong signal attributed to the P in the Ti sublayer is visible in the EDX mapping (Figure 8b). The numerical results are shown in Table 2.

**Figure 9:**
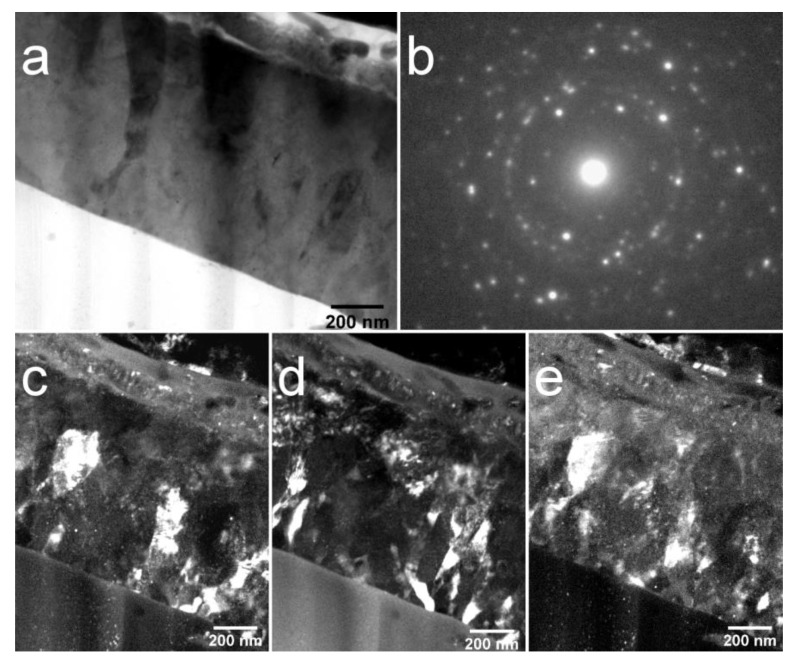
TEM image of the cross-section of Cu-HA coating on Ti after HT at 750 °C BF image (**a**), selected area electron diffraction (SAED) obtained from the coating region (**b**), DF in reflex (002) (**c**), DF in reflex (112) (**d**), and DF in reflex (213) (**e**).

In Figure 9a DF TEM image reveals a true size and shape of the Cu-HA coating grain structure. A dendritic type of grain growth is suggested. The SAED is represented by a series of concentric rings and bright reflexes confirming the polycrystallinity of the coating. The DF TEM image obtained in reflexes (002), (211), and (213) allowed to calculate the mean lateral size of grains that are varying from 30 to 60 nm. Hence, the shape and the size of grains formed during crystallization at 700 °C HT significantly differ from the ones obtained at 450 °C. The higher crystallization temperature resulted in grain growth towards multiple directions, a size reduction and a formation of interlayer are also distinctive features of crystallization at 700 °C HT. It is worth noting, that the grain arrangement and shape is similar to the structure of human teeth enamel, reported in the paper by A. Koblischka-Veneva et al. [54]. This scientific group reported that the thin structure of the enamel surface consists of a large number of crystallites with different orientations, but only a small range of grain sizes between 20 and 130 nm. The similar features could be found in the TEM results of Cu-HA coating on the Ti substrate obtained by us.

### 3.2. The Influence of Deposition at Increased Substrate Temperatures on Crystallization and Surface Morphology of Zn Containing CaP on Ti Substrate

In our previous report, the influence of substrate temperature on the formation of crystalline Zn-HA has been briefly discussed [55]. The proposed extended structure zone model [56,57] provides an insight into the crystalline structure formation during magnetron deposition as a function of adatom mobility. The main governing parameter in this model is energy available per arriving atom. This parameter should include not only the influence of the substrate temperature as was proposed before in classical Thorton’s model but also the kinetic energy of arriving atoms. In our work, we studied the influence of the substrate temperature on the formation of crystalline Zn-HA coating, when trying to keep the energy of the arriving atom at constant, by placing substrates in the projection area of the target erosion zone and using the same RF-power in all deposition runs.

**Figure 10:**
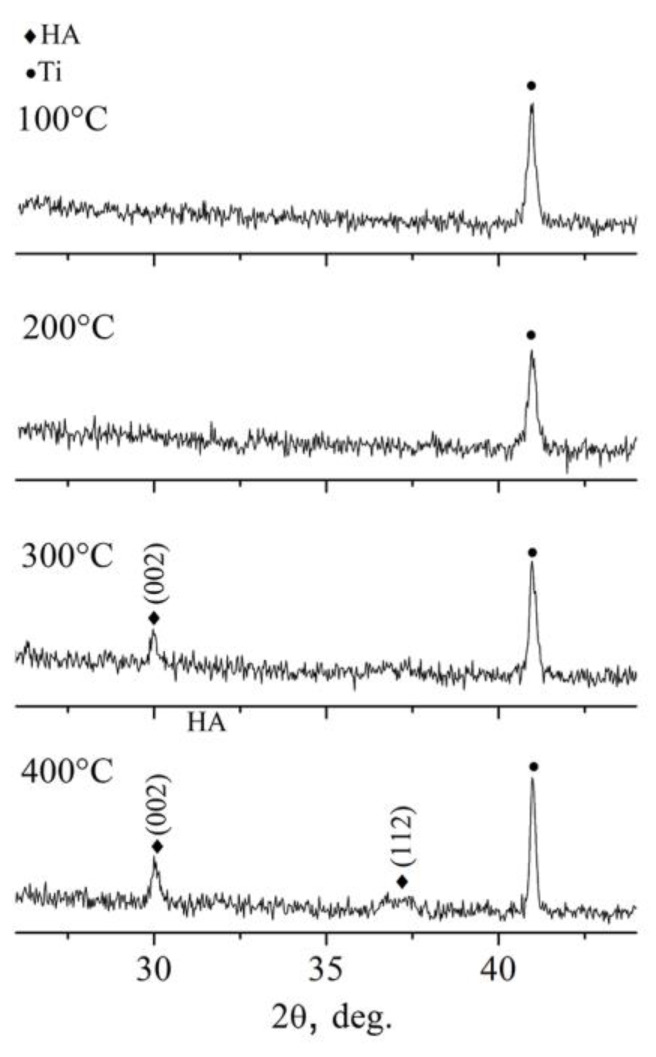
XRD profiles of Zn-HA coatings deposited at in situ substrate temperature 100 °C, 200 °C, 300 °C, and 400 °C.

As seen in Figure 10, at substrate temperatures below 200 °C Zn-HA coating remains X-ray amorphous. Only starting at the temperature of 300 °C does coating crystallizations occur. The preferential growth orientation of HA crystals is evident in the (002) plane. The lowest surface energy in the HA crystal, according to A. Ivanova et al. [58], is in the (002) plane. Hence, Zn-HA coatings tend to grow with a (002) orientation. According to XRD results (Figure 10), the preferential growth in (002) plane is more pronounced at the temperature of 400 °C and additional growth orientation for Zn-HA crystals in the (112) plane also could be detected. From that, we conclude that in order to deposit the Zn-HA coatings in a crystalline state the substrate temperatures should not be lower than 300 °C.

**Figure 11:**
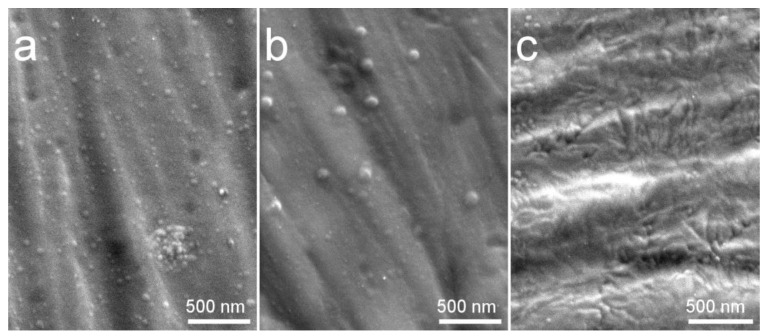
SEM images of Zn-HA coatings deposited at in situ substrate temperature 100 °C (**a**), 300 °C (**b**), and 400 °C (**c**).

In Figure 11, SEM images of coatings deposited at in situ substrate temperature of 100 °C (a), 300 °C (b), and 400 °C (c) are represented. Upon crystallization, the morphology of the coating changes from an increased number of dome-like features, previously observed in the case of amorphous Cu-HA deposited onto cold Ti and Mg0.8Ca substrates, to the morphology that predominantly consists of elongated elliptical grains. Multiple grain boundaries appeared upon crystallization at 400 °C. The same character of reduction of surface spherical clusters and their change to the grain-like spherical-free surface upon crystallization is observed. It appeared that upon crystallization amorphous globular domains firstly agglomerated in a bigger sized dome-like surface features at the substrate temperature of 300 °C (Figure 11b) later to be crystallized at 400 °C (Figure 11c). In all deposition cases, the surface of the coating is dense and crack-free, which is observed microscopically and by a simple visual appearance.

**Figure 12:**
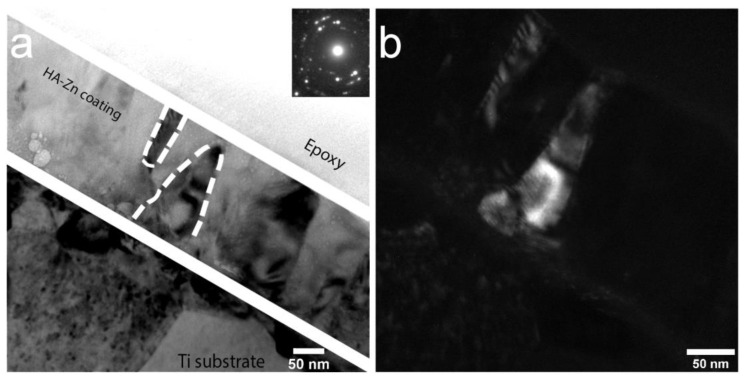
TEM image of the cross-section of Zn-HA coating on Ti deposited at the substrate temperature of 400 °C BF, the inset shows the corresponding electron diffraction pattern (**a**) and DF (**b**).

TEM analysis of the Zn-HA coating deposited at 400 °C substrate temperature was employed. In Figure 12a polycrystalline Zn-HA coating is revealed. In the DF TEM image (Figure 12b) the columnar character of growth is confirmed, evidenced by elliptical grains extending along with the whole coating thickness. The growth direction exhibits a strong orientation that is directed perpendicular to the substrate surface. The grains appear to be densely packed without any visible amorphous phase between each other. The represented in Figure 12b DF TEM image is obtained from the (112) reflex in SAED pattern of the Zn-HA coating having a high intensity. Note, the grains showed by the white dashed line in the bright-field image. Those grains are revealed to be consisting of sub-grains with distinct grain borders in the dark-field image as was also seen for Cu-HA coatings after HT. The amorphous TiO_2_ interlayer is visible in the micrograph.

**Figure 13:**
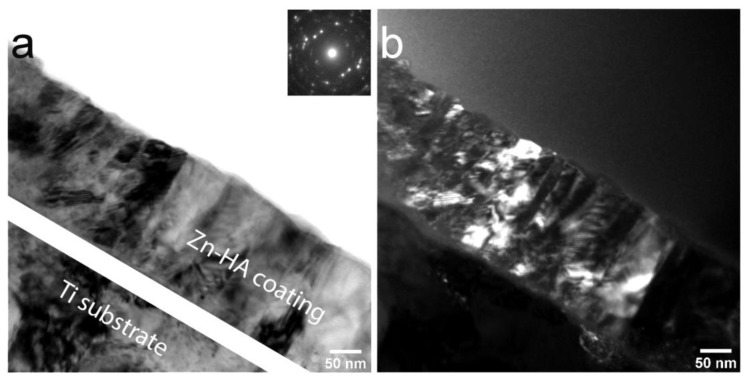
TEM image of the cross-section of Zn-HA coating on Ti deposited at the substrate temperature of 400 °C BF, the inset shows the corresponding electron diffraction pattern (**a**) and DF (**b**).

In the work by Ivanova et al. [58], crystalline HA coating was deposited at the substrate temperature not exceeding 300 °C with a formation of V-shaped columns as evidenced by TEM. In some regions of the lamella, the authors indicated the ACP interlayer. In our case, however, during the coating deposition at 400 °C the fraction of ACP is not pronounced and sporadically visible in local regions. In Figure 13a, the dense, polycrystalline structure of Zn-HA is confirmed. The coating thickness estimated from the TEM images is 140 ± 20 nm. The coating has a preferential orientation and is represented by a zoned structure exhibiting grains and sub-grains. Occasionally, Moiré pattern is visible in the DF TEM image, resulting from the rotation of different crystals in the structure of the coating. The average grain lateral size is ranging from 30 to 90 nm that was calculated from multiple regions across the lamella.

In-column EDX mapping was performed from the cross-sectional view between the crystalline Zn-HA and Ti substrate. Due to the relatively small concentration of Zn in the HA structure, an EDX analysis was not able to reliably detect this element. The average Ca/P ratio calculated from the coating region was found to be equal to 1.5. Hence, the Zn-HA coating deposition at 400 °C substrate temperature leads to the formation of sub-stoichiometric HA. Both Zn substitution and preferential phosphate groups desorption from the coating during condensation under elevated substrate temperatures could play a role in the existing elemental composition.

**Figure 14:**
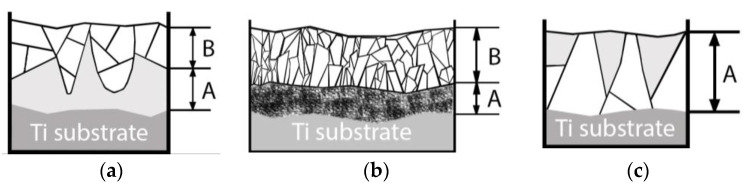
Schematic illustration of CaP microstructure after HT at 450 °C (**a**), HT at 700 °C (**b**), and in situ substrate heating to 400 °C (**c**).

Lastly, a schematic microstructure evolution of CaP coatings based on the performed TEM analysis is represented in Figure 14.

In the case of post-deposition HT in the Ar atmosphere, a crystallization is guided by a temperature gradient that is, in turn, dependent on the thermal conductivity of the coating and substrate material. Thermal conductivity (k) of HA = 0.16 W/(m K) [59], for commercially pure Ti = 16.3 W/(m K) [46,60], and for Mg0.8Ca = 130 W/(m K) [61]. During a post-deposition, HT crystallization starts from the top side of the coating and slowly propagating towards the substrate as it is depicted in Figure 14a. While the heat was dissipating through the metallic substrate from one side, the crystallization already starts from the coating top side resulting in the growth of V-shaped grains in an amorphous matrix. The majority of the crystalline grains consist of sub-grains. The amorphous interface between the crystalline part and the surface of the substrate is always present at the 450 °C HT. In case of the HT at 700 °C, more rapid crystallization occurs (Figure 14b). In this case, crystallites grow in extremely competitive conditions which results in finer, more elongated grains. A higher fraction of grains is represented by elliptical shapes with sharp, pointy edges, contrary to the more rounded shape seen before for grains formed at a temperature of 450 °C. A dendritic type of growth is suggested for the coatings obtained during 700 °C HT. Moreover, a distinct interlayer between the substrate surface and the columnar crystalline part is formed. This layer consists of fine phosphorous—poor equiaxed grain structure. It could be speculated that this interlayer was formed upon relatively fast cooling in the condition of drastically different heat conduction of HA and Ti material. Thus, we observed two distinctly different results of post-deposition HT. Lastly, during the deposition of the Zn-HA coating on the substrate maintained at 400 °C, an inversed V-shaped grain structure in the amorphous matrix is evident (Figure 14c). Here we see that the lateral size of crystallites is the highest close to the interface between the substrate and the coating, an inverse to what was previously observed for post-deposition HT at 450 °C. Even though a relatively small fraction of ACP is present, the coating structure is mostly consisting of crystallites propagating through the whole coating thickness. Due to the small concentration of doping elements such as Cu or Zn, the same crystallization behavior is expected for pure HA. In Table 3, a summary of the surface morphology, structure, crystallite size calculated from the TEM images and elemental composition according to the in-column EDX of deposited coatings at the different deposition regimes and post-deposition HT are represented.

## 4. Conclusions

The effect of post-deposition heat treatment vs. coating deposition at increased substrate temperatures on crystallization and surface morphology of the Cu and Zn containing CaP on Mg–Ca and Ti alloys deposited by an RF magnetron sputtering was studied. The character of the crystalline structure is not only governed by the temperature itself but relies on the condition either post-deposition treatment, where the ACP undergoes crystallization or instantaneous crystalline coating growth during deposition on the hot substrate. It is established, that the crystalline structure obtained during post-deposition heat-treatment of ACP on Ti or Mg–Ca substrate is distinctively different upon applied treatment temperature. The higher the treatment temperature, the finer size crystallites that are formed. It is possible to form a crack and void-free crystalline CaP coating on the surface of Mg–Ca alloy by heat-treatment at 450 °C for 3 h in Ar atmosphere. Higher temperature heat-treatment at 700 °C results in local coating micro-cracking and induced defects. The surface morphology in the case of as-deposited X-ray amorphous CaP consists of globular-like fine surface ACP clusters in both cases of Cu-HA and Zn-HA sputtering targets. Upon crystallization, either by post-deposition heat-treatment or deposition at increased substrate temperature at 400 °C and higher, ACP clusters agglomerate and crystalize significantly alter the surface of a CaP coating. Thin structure evolution scheme during the increase in heat-treatment temperature in relation to the structure of deposited at increased substrate temperature coating is purposed.

## Figures and Tables

**Table 1:** Calculated values for hydroxyapatite structural parameters (*a* and *c*—lattice parameters).

Sample	*a* (nm)	*c* (nm)	Δ*d/d*	Average Crystallite Size (CSR) (nm)
Cu-HA coating on Mg0.8Ca after 450 °С HT for 3 h	0.9444	0.6947	2.9 × 10^−3^	37 ± 15
Cu-HA coating on Ti after 450 °С HT for 3 h	0.9547	0.686	3.1 × 10^−3^	47 ± 17
Cu-HA coating on Ti after 700 °С HT for 3 h	0.9534	0.6783	3.3 × 10^−3^	13 ± 9
Cu-HA target	0.9386	0.6895	-	-
Data according to ICDD	0.9422	0.6881	-	-

**Table 2:** Elemental composition of the Cu-HA coating after HT at 700 °C.

Region of Detection	Elemental Composition (at.%)		Ca/P (at.%) Ratio
	Ca	P	
Crystalline	16.98 ± 3.84	10.11 ± 1.11	1.69
Interlayer	13.69 ± 0.45	0.74 ± 0.1	18.5
Ti sublayer	0.5 ± 0.1	1.67 ± 0.05	0.3
Mean value gathered from multiple regions of Cu-HA coating	14.91 ± 2.37	7.48 ± 0.86	2.0

**Table 3:** Comparison table of deposited coatings.

Target Material	Substrate Material	Temperatur (°C)	Surface Morphology	Structure	Crystallite Size (nm)	Concentration (at.%)		Ca/P (at.%) Ratio
						Ca	P	
Cu-HA	Ti	<100	Dense and void-free with dome-like surface features of 10–60 nm size	Amorphous state	-	17.23 ± 1.75	9.74 ± 0.72	1.77
	Mg0.8Ca							
	Ti	450	Dense and void-free with sharp crystallites	Crystalline state, V-shaped growth in the ACP matrix	45–100	16.57± 1.32	8.94± 0.85	1.85
	Mg0.8Ca							
	Ti	700	Occasional micro-cracking, the surface is comprised of sharp crystallites of nm	Crystalline state with dendritic type of structure and formation of the interlayer	20–130	14.91 ± 2.37	7.48 ± 0.86	2.0
Zn-HA	Ti	100	Dense and void-free, dome-like features of 15–80 nm	Amorphous state	-	-	-	-
	Ti	200	Dense and void-free, dome-like features	Amorphous state	-	-	-	-
	Ti	300	Dense and void-free, dome-like features with agglomerates of 35–140 nm	Start of the crystalline growth	-	-	-	-
	Ti	400	Dense and void-free, surface grains are changed to an elongated elliptical shape	Crystalline state, Λ-shaped growth in ACP matrix	30–90	12.97 ± 1.76	8.71 ± 0.91	1.5
